# Mesenteric cystic lymphangioma in an adolescent male; a diagnostic dilemma: A case report

**DOI:** 10.1016/j.ijscr.2023.109042

**Published:** 2023-11-14

**Authors:** Asmita Bhusal, Quazi Habibullah, Mashiur Rahman, Biplob Bandh, Saiful Islam, Tanvin Dola, Saugat Bista

**Affiliations:** a Sir Salimullah Medical College and Mitford Hospital, Dhaka, Bangladesh; b Department of Surgery, Sir Salimullah Medical College and Mitford Hospital, Dhaka, Bangladesh; c Sir Salimullah Medical College, Dhaka, Bangladesh

**Keywords:** MCL, Hemangioma, Hemolymphangioma, Adolescent tumor, Surgical resection, Case report

## Abstract

**Introduction and importance:**

Hemolymphangioma, a rare type of lymphangioma, is a benign hamartoma of the blood vessels and lymphatic system. Considered to be extremely rare among adults with infrequent occurrence in abdominal regions, lymphangioma involving mesentery accounts for <1 % of cases and only 0.05 % involve the GI tract. Due to the absence of typical clinical presentation, making a confirmatory preoperative diagnosis is challenging.

**Case presentation:**

An 18-year-old Bangladeshi man visited the hospital with the complaints of epigastric and umbilical pain for 15 days which was insidious in onset. Physical examination revealed an ill defined lump that was palpable on the right side of the abdomen. Ultrasonography showed multiloculated cystic mass having septation approximately 13 × 6 cm in size. An abdominal CT scan showed cystic lesion with septations measuring about 14.5 × 12.3 cm, compressing the bowel loops towards left, that was suggestive of mesenteric lymphangioma. The patient underwent partial surgical resection. The excised mass was sent for histopathology. Histopathology disclosed a benign tumor composed of proliferation of blood vessels of different sizes lined by endothelium in a fibromuscular stroma.

**Clinical discussion:**

Mesenteric lymphangiomas are infrequent malformations and very few cases of mesenteric hemolymphangioma have been reported in adults. Histopathology is required for confirmatory diagnosis and immunohistochemistry is required to differentiate the tumor subtype. Surgical resection is deemed a standard treatment modality.

**Conclusion:**

We report an extremely rare case of mesenteric hemolymphangioma to bring it to concern that even with the vague clinical presentations and involvement of age groups beyond the status quo, surgeons must be vigilant about abdominal MCL/hemolymphangioma and proceed accordingly.

## Highlights

• Cystic lymphangioma rarely involves the abdominal regions and the majority of those involving mesentery become symptomatic before the age of 2.
• We report an unusual case of surgically confirmed large hemolymphangioma in an 18-year-old Bangladeshi man.
• Preoperative diagnosis and treatment plan relies on radiological imaging as establishing clinical suspicion is highly challenging due to the lack of typical presentations.
• Since confirmatory diagnosis is reached through a postoperative histopathological examination and immunohistochemistry, financial limitations and lack of availability of high-end diagnostic techniques can act as a barrier to establish the same.
• Laparotomy followed by partial surgical resection was carried out as a standard treatment modality.
• A thorough follow-up following surgery is necessary to detect any signs of recurrence.

## 1 Introduction

Mesenteric cystic lymphangioma are infrequent benign malformations of the lymphatic vessels which rarely occur in the abdominal regions. Cystic lymphangiomas in adults make up 7 % of abdominal cysts [1]. Of all abdominal lymphangiomas, <1 % involve the mesentery and 90 % of these develop symptoms before the age of 2 [2]. Their clinical presentations are neither suggestive nor very specific and may be different based on their location and size of the cysts [3]. The most common site of involvement of cystic lymphangiomas include face, neck, axilla and thoracic regions. GI tract involvement accounts for <0.05 % of such tumors [4].

In this, we report a rare case of surgically confirmed large hemolymphangioma in an adolescent male which presents its importance in highlighting the diagnostic limitations regarding the case in an economically disadvantaged society where accessibility to high-end diagnostic techniques even in a tertiary level hospital is not a given and hence, raising awareness to be vigilant regarding the workup in such a setting. It also puts forth a great message in the realm of surgical faculty that without prior experience of a rare case as such, the diagnostic suspicion is less likely to build as it was the first ever case of mesenteric hemolymphangioma that the surgical team dealt with in our medical college and hospital. This report has been written in line with the SCARE criteria [5].

## 2 Case presentation

An 18-year-old young man was admitted with the complaints of epigastric and umbilical pain for 15 days that was insidious in onset and dull aching in nature. The intensity of the pain was mild to moderate. The pain was not related to any specific aggravating factor including food intake. Pain was relieved by taking oral medications. He noticed a gradual increment in the intensity of pain for 7 days. He also complained of fever during the last 7 days that was continuous in nature. It was not associated with chills and rigor. The highest temperature recorded was 101 degree Fahrenheit that decreased with the intake of antipyretic. He did not recall vomiting and did not experience weight loss, cough, hematemesis, malena and jaundice. His bowel and bladder habit was normal. There were no major ailments in the past. He had no medical history of abdominal trauma or surgery and no family history of malignant tumor. He belongs to a poor socio- economic background and maintains satisfactory personal hygiene.

On physical examination, the patient was ill-looking and co-operative with a BMI of 19.92 kg/m^2^. He presented with an increased body temperature, anemia was not evident, he was non icteric, Virchow's gland and accessible lymph nodes were impalpable. On inspection, the abdomen was flat with centrally placed, inverted and vertically slitted umbilicus, and intact hernial orifices. There was no visible swelling and peristalsis. On palpation, the abdomen was mildly tender over the epigastrium, umbilicus, right hypochondriac and right lumbar region. An ill-defined intra abdominal lump was found involving the right hypochondriac and lumbar region which had a smooth surface, was soft in consistency, partly mobile from side to side but not above downwards, non-pulsatile and not moving with respiration. On auscultation, bowel sound was present which was normal. No bruit was audible. Digital rectal examination revealed no abnormalities. No other systemic abnormalities were observed.

After a thorough history-taking and examination, we proceeded for laboratory investigations, imaging, and histopathology as a part of diagnostic workup. No abnormality was detected in laboratory investigations including Complete Blood Count (CBC), Serum amylase, lipase, electrolytes, creatinine, and Random blood sugar (RBS). Echinococcal antibody was also negative. Table 1.

**Table 1:** Laboratory tests.

Test	Value	Normal range and values
Complete Blood Count (CBC)		
Hb%	14.2 g/dl	M: 13–18, F: 11.5–16.5 g/dl
ESR	22 mm in first hour	M: 0–10, F: 0–20 mm/1st hr
Platelet count	447,000/cumm	150,000-450,000/cumm
WBC	9800/cumm	4000–11,000/cumm
Serum amylase	65 U/l	28–100 U/l
Serum lipase	82 U/l	0–160 U/l
Serum creatinine	0.8 mg/dl	0.6–1.3 mg/dl
Random Blood Sugar (RBS)	4.9 mmol/l	<7.8 mmol/l
Serum electrolytes		
Na	142 meq/l	135–146 meq/l
K	4 meq/l	3.5–5.3 meq/l
Cl	107 meq/l	97–106 meq/l
Echinococcal antibody	Negative	

**Fig. 1:**
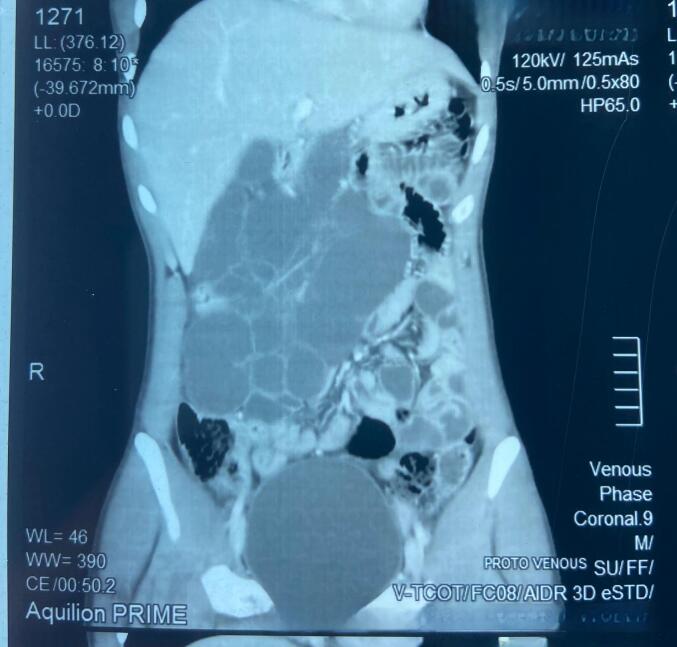
Contrast enhanced CT scan of whole abdomen: Cystic lesion with septations noted at right side of abdomen, measuring about 14.5 ∗ 12.3 cm, compressing the bowel loops towards left. Peripheral & septal enhancement noted after IV contrast. No evident solid component detected. No abnormality detected in liver, gallbladder, pancreas and kidneys. Comment: CECT suggestive of Mesenteric lymphangioma.

X-ray abdomen was found to be normal. Ultrasonography (USG) showed multiloculated complex cystic mass having septation approximately 13 × 6 cm in size on the right side of the abdomen. We then carried out ultrasonography guided fine needle aspiration cytology of the abdominal mass which showed smears with lymphocytes and foamy histiocytes in a background of thick proteinaceous material. No evidence of malignancy was seen and the findings were compatible with lymphangioma. Contrast enhanced Computed Tomography (CECT) scan was performed and the findings of CECT were consistent with cystic lesion with septations noted at right side of abdomen, measuring about 14.5 ∗ 12.3 cm, compressing the bowel loops towards left. After IV contrast it showed peripheral and septal enhancement. No evident solid component was detected. It showed no abnormality in liver, gallbladder, pancreas and kidneys and was suggestive of mesenteric lymphangioma. Origin of the lesion could not be mentioned (Fig. 1).

**Fig. 2:**
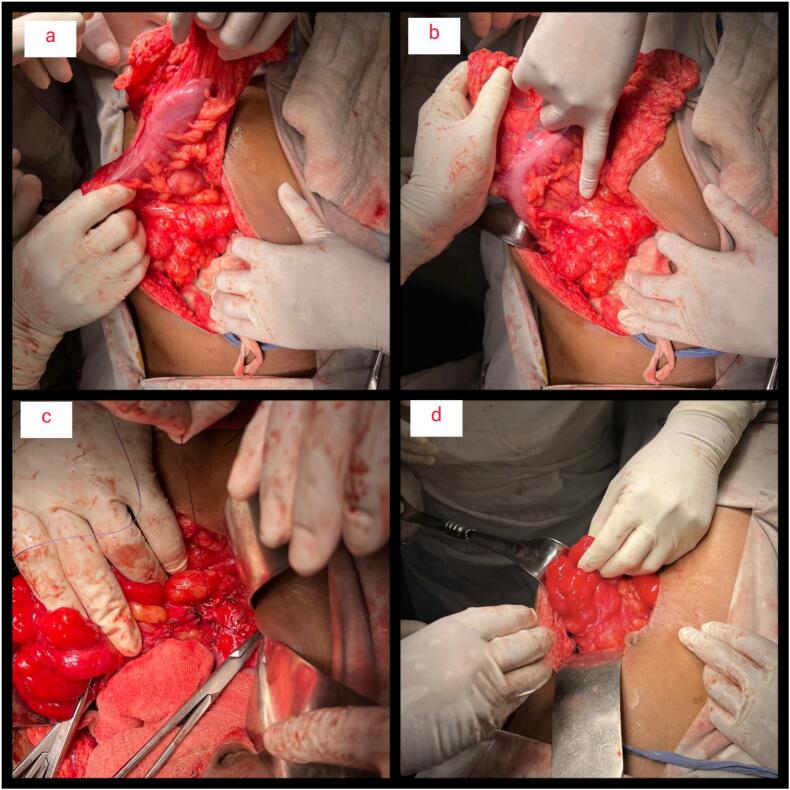
(a,b,c,d) Intraoperative findings: Following an upper midline incision, a large cystic multiloculated lesion was found on the right side of the infracolic compartment. The base of the lesion measured about 10 × 10 cm and involved the small bowel mesentery near the superior mesenteric vessels. The lesion was adherent with transverse colon, duodenum, and small gut (i.e. jejunum).

Following the challenging diagnostic workup, with the patient's informed written consent we concluded the surgical approach to be our definitive modality of treatment. We conducted a thorough preoperative assessment to prepare the patient for surgery. Thereafter, laparotomy followed by partial surgical resection of the mesenteric lymphangioma was carried out along with adhesiolysis and hemostasis. The margin at the base of the lesion could not be cleared. Patient had been placed under general anesthesia with endotracheal intubation in supine position (Fig. 2a,b,c,d). The specimen was resected and it was sent for histopathology. A gross overview of which showed specimen consisting of an irregular gray brown cystic structure measuring about 12 ∗ 6.5 ∗ 1.5 cm. Cut surface revealed mostly cystic and partly solid structure. Cut section showed brownish fluid. 3 lymph nodes were also separated from the specimen. The microscopic examination of the sections made from the submitted specimen exhibited a benign tumor composed of proliferation of blood vessels of different sizes lined by endothelium in a fibromuscular stroma. It also revealed areas of hemorrhage. The lymph nodes that were separated revealed follicular hyperplasia. No granuloma or malignancy was evident. Thus, the histopathological findings were compatible with hemangioma.

**Fig. 3:**
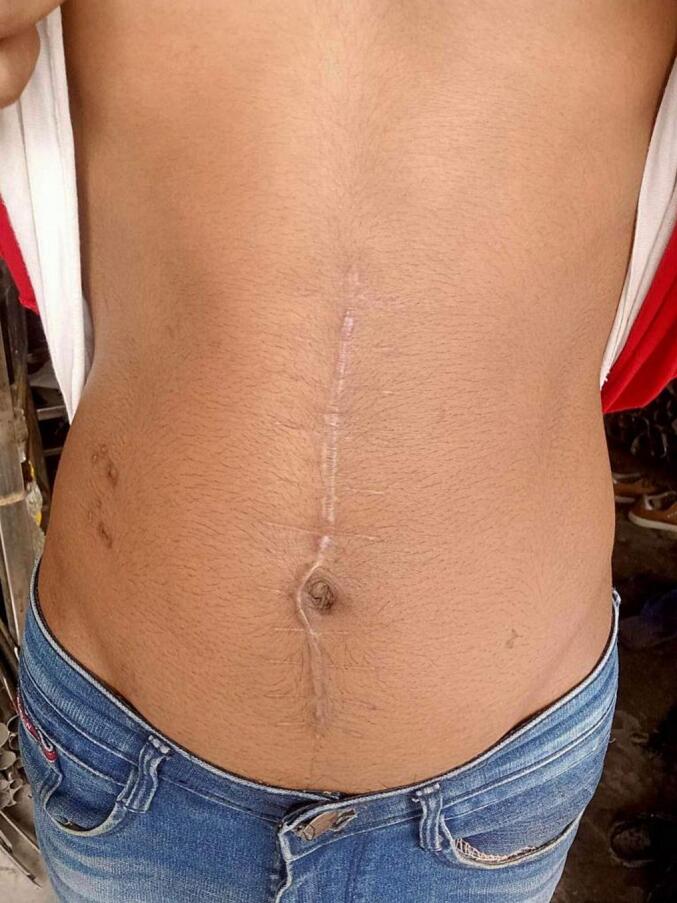
Clinical picture taken 2 months after surgery.

Our patient had a smooth surgical recovery devoid of any complications and a follow-up after two months of surgery revealed no signs of recurrence (Fig. 3).

## 3 Discussion

Mesenteric lymphangiomas are infrequent congenital malformations that manifest as a non-epithelial, neoplastic proliferation of lymphatic channels [6]. The definitive diagnosis is based on histologic characteristics, which use the depth and size of the abnormal lymphatics to identify four tumor subtypes that are as follows: cavernous, capillary, cystic hygroma, or hemolymphangioma (i.e. mixed vascular/lymphatic elements) [7]. The postoperative findings of our patient corresponded with the latter. As easy as concluding the diagnosis in one mere statement might seem, in retrospect the diagnostic workup was highly challenging due to financial constraints given the poor economical background of the patient and the limited availability of diagnostic tools and resources in our medical college and hospital.

Hemolymphangioma, originating from the mesenchymal tissue, is a benign hamartoma of the blood vessels and lymphatic system [8]. This is a rare type of lymphangioma, and it appears to have a mixture of blood and lymphatic vessels [9]. Hemolymphangioma can be classified into two types i.e. primary and secondary. A primary lymphatic vascular tumor is considered to be a congenital malformation of the lymphatic vascular system, probably caused by a blockage in veno-lymphatic communications between the dysembryoplastic vascular tissue and systemic circulation. On the other hand, secondary arises from insufficient lymph drainage and lymphatic damage caused by surgery or trauma [8,10]. In our case, the patient had no previous history of abdominal trauma or any intra abdominal surgery, making it rare and difficult to categorize in terms of causation. Clinical manifestations of abdominal hemolymphangioma are likely to vary depending on their location and size [11]. The symptoms differ according to the pressure exerted on surrounding structures, which can be attributed to an enlarging mass or complications like hemorrhage, infection, torsion, perforation, and rupture [12].

In case of large abdominal hemolymphangioma, patients may present with acute abdominal pain and discomfort, and bowel obstruction [9]. Clinical settings in which patients present with features like weight loss, loss of appetite, postprandial fullness and abdominal pain mimicking malignancy is yet another misleading scenario as evident in a previously reported case of a 27-year-old male patient who was diagnosed with an ileal MCL [13]. Abdominal pain is the most common clinical presentation [14]. As seen in our case, the patient presented with abdominal pain, fever, and an ill-defined abdominal lump which led us to suspect a number of differential diagnoses. We proceeded keeping in mind that the exclusion of the differentials might help us reach a confirmatory diagnosis. Differential diagnoses in our case based on the clinical presentation were: infected mesenteric cyst, hydatid cyst, pancreatic pseudocyst, and liver abscess. From the findings of laboratory investigations like echinococcal antibody that was found to be negative, normal levels of serum amylase and lipase, and normal imaging studies of the suspected visceral organs in X-ray abdomen, USG, and Computed Tomography/CECT, we could successfully exclude all other differentials allowing us to proceed with mesenteric cystic lymphangioma in mind. However, we were not aware of the fact that there could be a massive discrepancy between the preoperative radiological diagnosis and postoperative histopathology findings as this was the very first case of mesenteric hemolymphangioma in our hospital. Accurate preoperative diagnosis of intestinal or mesenteric hemolymphangioma is very difficult as shown by previously reported cases [9]. Since there are no typical clinical symptoms in hemolymphangioma, we have to rely on radiological imaging such as CT and Magnetic Resonance Imaging (MRI) to make a preoperative diagnosis and formulate a treatment plan [16]. Although radiological imaging can evoke a preoperative diagnosis, it can be confirmed only after a postoperative histopathological examination [15].

In particular, specific enhanced characteristics can be observed on imaging with regard to the differing size of blood vessels in hemolymphangioma [16]. In tumors that are rich in blood vessels there may be significant and persistent enhancement; in the meantime, there may be marked enhancement in the septum too [17,18]. In our case too, CT showed peripheral and septal enhancement after contrast. As a result of the capillary permeability of the contrast agent molecules, intravenously administered CT contrast agents like iodine rapidly diffuse into the extravascular space, which is why the extravascular interstitial space gets enhanced [9]. The majority of intestinal hemolymphangioma was found to be solid [19,20] and a purely cystic case has also been reported [16,21]. Our case presented as a huge cystic lesion since no solid component was evident even in CECT (contrast-enhanced computed tomography). It is suspected that the proportion of vascular and lymphoid components, presence or absence of degeneration, may alter the individual imaging features of hemolymphangioma which ranges from pure cystic to solid [9]. Surgical resection is the standard management of hemolymphangioma [11]. Our patient underwent a partial surgical resection of the mass as the margin of the base could not be cleared. Intraoperatively, the lesion was found to be a multiloculated cyst that arose from the mesentery and involved other parts of the GIT. From the surgical specimen, thick clear fluid with minimal hint of hemorrhage spilled out.

According to the imaging findings, we presumed it to be a case of mesenteric cystic lymphangioma but later postoperative histopathological examination showed hemorrhagic components compatible with a hemangioma. Since the radiological findings in our case was not typical of a hemangioma, the discrepancy between the imaging findings suggestive of a lymphangioma and surgical pathology reports compatible with hemangioma could have been eliminated through immunohistochemistry (IHC). In contrast, the poor socioeconomic background of the patients can act as a hindrance in opting for expensive diagnostic evaluations in case of third world countries such as Bangladesh. Given the underprivileged social position of our patient and lack of availability of high-end diagnostic techniques in our medical college and hospital, we could not carry out an immunohistochemistry examination.

Overall, it may be possible to achieve the best results with a reduced recurrence rate by complete excision; however, thorough follow up is required [19]. There have been reports in the literature of a 10–27 % recurrence rate for lesions after complete surgical resection, whereas 50–100 % of partially resected tumors can recur [19]. Our patient was in a good state after surgery, and there were no signs of recurrence as established by a follow up at 2 months of surgery.

## 4 Conclusion

We report an extremely rare case of mesenteric hemolymphangioma to bring it to concern that even with vague clinical presentations and involvement of age groups other than the usual majority as evident in this case, surgeons must not completely exclude the suspicion of abdominal MCL/hemolymphangioma as a differential and should proceed accordingly.

Similarly, we aim to highlight the importance of roles of imaging techniques in accurate preoperative diagnosis and surgical planning. Patients will be highly benefited if radiologists are more aware of the radiological findings of MCL/hemolymphangioma especially in an economically disadvantaged social setting where patients can't afford expensive diagnostic tests like immunohistochemistry.

Having said that, the requirement of histopathology in making a confirmatory diagnosis and immunohistochemistry to differentiate the tumor subtype should also be given the utmost importance while managing such cases.

## Consent

Written informed consent was obtained from the patient for publication of this case report and accompanying images. A copy of the written consent is available for review by the Editor-in-chief of this journal on request.

## Provenance and peer review

Not commissioned, externally peer-reviewed.

## Ethical approval

Nothing to declare.

## Funding

The author(s) received no financial support for the research, authorship and/or publication of this article.

## Author contributions

Dr. Asmita Bhusal was involved in the ideation, data collection, drafting, designing and editing of the manuscript, and took entire responsibility for publishing this study under the supervision of Dr. Quazi Habibullah and Dr. Mashiur Rahman.

Dr. Quazi Habibullah was the chief surgeon of the case.

Dr. Mashiur Rahman and Dr. Biplob Bandh reviewed the manuscript.

Tanvin Dola, Dr. Saiful Islam and Dr. Saugat Bista helped in writing the manuscript.

All the authors read the paper and gave final approval of the version to be published.

## Guarantor

Dr. Asmita Bhusal and Dr. Quazi Habibullah accept full responsibility for the work and/or the conduct of the study, had access to the data, and controlled the decision to publish.

## Research registration number

Not applicable.

## Conflict of interest statement

None to declare.
